# The effects of formalized and trained non-reciprocal peer teaching on psychosocial, behavioral, pedagogical, and motor learning outcomes in physical education

**DOI:** 10.3389/fpsyg.2015.00149

**Published:** 2015-02-17

**Authors:** Peter R. Whipp, Ben Jackson, James A. Dimmock, Jenny Soh

**Affiliations:** School of Sport Science, Exercise and Health, The University of Western AustraliaPerth, WA, Australia

**Keywords:** unidirectional teaching, motivation, motor performance, learning climate, tactical games approach

## Abstract

Peer teaching is recognized as a powerful instructional method; however, there is a paucity of studies that have evaluated the outcomes experienced by peer-teachers and their student recipients in the context of trained, non-reciprocal, high school physical education (PE). Accordingly, the effectiveness of a formalized and trained non-reciprocal peer teaching (T-PT) program upon psychosocial, behavioral, pedagogical, and student learning outcomes within high school PE classes was investigated. Students from eight intact classes (106 males, 94 females, *M_age_* = 12.46, *SD* = 0.59) were randomly assigned to either a T-PT intervention group (taught by a volunteer peer-teacher who was trained in line with a tactical games approach) or untrained group (U-PT; where volunteer peer-teachers received no formal training, but did receive guidance on the game concepts to teach). Data were collected over 10 lessons in a 5-week soccer unit. Mixed-model ANOVAs/MANOVAs revealed that, in comparison to U-PT, the T-PT program significantly enhanced in-game performance actions and academic learning time among student recipients. Those in the T-PT also provided greater levels of feedback and structured learning time, as well as reporting more positive feelings about peer teaching and fewer perceived barriers to accessing learning outcomes. These findings show that non-reciprocal peer-teachers who receive formalized support through training and tactical games approach-based teaching resources can enhance behavioral, pedagogical, and motor performance outcomes in PE.

## INTRODUCTION

School-based physical education (PE) plays an important role in children’s physical growth and development, and is an integral part of a school curriculum. Through health and PE, students are able to acquire the knowledge, skills, attitudes and values that facilitate the pursuit of a physically active and healthy lifestyle (Siedentop and Tannehill, 2000). Hence, researchers and PE teachers are continually exploring teaching strategies in order to meaningfully engage and motivate students to achieve learning outcomes (Whipp et al., 2012). Of concern, some PE teachers equate teaching effectiveness solely with well-mannered (i.e., good), well-occupied (i.e., busy), and satisfied students (Placek, 1983; Schempp, 1985). Although these factors are important, other aspects relating to student learning, lesson structure, student engagement, and lesson variety are also critical, and these aspects have received less attention (Silverman, 1991). Teaching strategies, namely the approaches used by teachers for instruction, represent one key factor that has a direct influence on student learning. The National Association for Sports and Physical Education [NASPE] (1995), promoted peer teaching as an educationally appropriate strategy to enhance student learning. Moreover, Siedentop and Tannehill (2000) proposed peer assisted learning as a best practice to influence student learning outcomes.

Peer teaching is a student-centered instructional technique (Mosston and Ashworth, 1985, 2002) that situates the students as the deliverer of direct instruction (Rosenshine, 1979). Within this instructional format the student provides a peer or peers information about task performance in a cooperative manner to help them learn. Approaches to peer teaching include teaching in pairs (Mosston and Ashworth, 2002; Byra, 2006), class-wide peer tutoring (Johnson and Ward, 2001), peer-mediated accountability (Ward et al., 1998), peer assessment (Whipp et al., 2012), as well as structured and sequenced collaboration such as cooperative learning strategies to achieve a common goal (Cohen, 1994; Ward and Lee, 2005). In a peer teaching approach, students themselves are held responsible for the acquisition of knowledge, performance, and social skills, and are encouraged to achieve these outcomes via the development of positive interdependence, individual accountability, face-to-face interaction, and interpersonal skills (Cohen, 1994; Antil et al., 1998; Putnam, 1998; Dyson and Grineski, 2001). The effectiveness of peer teaching is dependent upon a complex set of factors that include students’ age and ability, motivation to collaborate, the nature of the task, the institutional and cultural support (Hogan and Tudge, 1999), as well as the competence level of the peer-teachers (Houston-Wilson et al., 1997; d’Arripe-Longueville et al., 2002). Importantly, in published PE-based peer teaching investigations, researchers have reported improvement in motor skill performance (Houston-Wilson et al., 1997), academic learning time (dePaepe, 1985; Webster, 1987), and moderate-to-vigorous physical activity levels (Lieberman, 2000). That said, a large proportion of existing peer teaching studies have been conducted in special educational settings (e.g., children with disabilities and/or additional learning needs), and may not generalize well to all PE classes.

The overarching aim in this investigation was to explore the effectiveness of a formalized non-reciprocal peer teaching program, taught by trained teachers, in relation to students’ psychosocial (enjoyment, autonomy support and motivation), behavioral (engagement level in physical activity), pedagogical (academic learning time, provision of feedback and structured learning time), and motor performance (game tactics in soccer) outcomes. Non-reciprocal peer teaching is unidirectional, where a student volunteer with pre-determined expertise and motivation, in consultation with the class teacher, adopts a teaching role to his/her peer/s. In the absence of any published specific nomenclature, peer teaching was operationalized in this study by: (a) providing peer-teachers with training in fundamental teaching methods (i.e., explanation of the PE Engagement and Reinforcing Model and provision of feedback; Soh, unpublished master’s thesis), and (b) providing peer-teachers with a resource package for each lesson taught that included detailed information about drills, question/answers and teaching cues based on the tactical games approach (Griffin et al., 1997; resource available on request from first author).

Enjoyment is an important psychological construct in understanding students’ physical activity experiences and motivation (Scanlan and Simons, 1992). There have been a number of studies that have associated PE enjoyment with the behavior of social agents such as peers (Carlson and Hastie, 1997) and teachers (Cai, 1998; Cecchini et al., 2001). Carlson and Hastie (1997), for example, found that peer affiliation was a primary source of PE enjoyment among Grade 8 and 9 students. In an investigation to examine students’ opinions of direct, peer, and inquiry teaching strategies, Cothran and Kulinna (2006) found that students rated the more social aspect of peer instruction as being the most fun and enjoyable way to teach PE. Although there has been considerable research on the psychological benefits of peer teaching, the majority of these studies have been focused on the impact upon student recipients, and comparatively few have explored the specific influence of peer teaching on the tutors’ (as well as recipients’) psychological responses (Legrain et al., 2003). Accordingly, in this investigation, we sought to explore the effects of a peer teaching training program (treatment/intervention) on the psychosocial outcomes reported by the recipients and delivers of the instruction.

While scant, there appears an emerging body of literature that supports the use of peer teaching structures with respect to PE student enjoyment, performance, and engagement (Byra, 2006; Ensergueix and Lafont, 2010; Iserbyt et al., 2011; Jenkinson et al., 2013). A significant proportion of PE-situated peer teaching studies have involved primary age students (Jenkinson et al., 2013). Only four studies have focused on PE- and motor-related outcomes in intact classes of secondary school students (Ernst and Byra, 1998; d’Arripe-Longueville et al., 2002; Ensergueix and Lafont, 2010; Iserbyt et al., 2011), and these studies incorporated reciprocal peer mediated instruction. Conversely, non-reciprocal peer teaching respects the need for and value of peer-teaching students’ possessing pre-developed pedagogical content knowledge through their own training and sporting experiences. As a consequence, they are potentially viewed, and hopefully respected, by the peer-participants as possessing sporting expertise.

Therefore, in this investigation the focus was on non-reciprocal (i.e., unidirectional) peer teaching in intact secondary school PE classes, a context which has had minimal attention. There is also currently no conclusive evidence regarding the optimum time needed to elicit positive peer teaching learning outcomes among student recipients. While enhanced motor skills have been recorded in short time-frames, including a one-off peer teaching session (d’Arripe-Longueville et al., 2002) and a 5-by-2-h lesson intervention (Ensergueix and Lafont, 2010), the approaches used in both studies were underpinned by reciprocal peer teaching, not non-reciprocal peer teaching in small groups. In order to examine PE-related psychosocial and learning outcomes over an extended period of time, a 10-lesson, 300 min peer teaching intervention was developed.

Whipp et al. (2012) found that the success of a peer-teacher swimming class was determined by the swimming ability of the peer-teachers, with higher ability swimmers providing a greater amount of feedback. Consistent with this, peer-teachers’ proficiency and knowledge have been shown to contribute to the success of the student recipient’s psychosocial and performance outcomes (Ernst and Byra, 1998; d’Arripe-Longueville et al., 2002; Ensergueix and Lafont, 2010). In line with the positive link to peer-teacher’s knowledge and proficiency, the effect of training peer-teachers has received some research interest in the literature (Ward and Lee, 2005). Although not reported in association with measures of participants’ pre-intervention skill level, dePaepe (1985) and Houston-Wilson et al. (1997) observed that trained peer-teachers influenced the performance of tutees more than untrained peer-teachers in an integrated PE class. Legrain et al. (2003) also found that training sessions improved a peer-teacher’s ability to teach (i.e., coaching skills scores); however, they did not examine whether these improvements translated into improved student learning, and their work was undertaken in a non-PE setting with college-aged students. Ensergueix and Lafont (2010) concluded that reciprocal peer teaching by trained peer-teachers realized superior outcomes for motor performance, when compared to peer-recipient students engaging in peer teaching with no training. Although the authors of these studies have demonstrated the benefits associated with peer-teacher training programs, they did not employ a non-reciprocal PE peer teaching approach with students in intact secondary PE classes, and as such the efficacy of this method, in comparison to that proposed, remains somewhat unclear.

Using what could be argued as a form of quasi-training, Iserbyt et al. (2011) investigated the effects of peer teaching in a high school (i.e., year 8) PE tennis setting using task cards to support instructions, feedback, and learning. Tennis performance was measured before and after a 4 week peer teaching intervention period, and results showed that peer-mediated learning with task cards was equally effective as teacher-centered instruction. Enhanced motor skill development has been reported elsewhere for PE-related peer teaching interventions for intact classes of swimming (d’Arripe-Longueville et al., 2002), table tennis (Ensergueix and Lafont, 2010), the forehand tennis stroke (Iserbyt et al., 2011) and the novice activity of juggling (Ernst and Byra, 1998). Despite an array of supporting evidence, Jenkinson et al. (2013) posited that (a) the effectiveness of peer teaching on performance has not been consolidated, (b) comparisons with other teaching strategies used to access learning outcomes are needed, and (c) skill enhancement in a discrete skill (e.g., a tennis serve) may not translate well to overall sporting performance.

In responding to the first of these concerns identified above, Iserbyt et al.’s (2011) task-card approach was the first study to show that peer-mediated learning was equally effective as an experienced qualified tennis teacher using a teacher-centered instructional format. Moreover, Iserbyt et al. (2011) reported that ALT-PE increased over the 4 weeks of their task-card PE peer teaching intervention, resulting in equal amounts and quality of ALT-PE in the intervention and teacher-centered control conditions. Iserbyt et al. (2011) also demonstrated that feedback rates were higher and instruction rates lower in a teacher-centered condition when compared to peer teaching. Iserbyt et al. (2011) were also the first to report on the rate or form of feedback in a peer-mediated PE intervention. Although they observed peer-teachers providing less feedback moments and lower technical feedback rates when compared to an instructor in a teacher-centered condition, they also identified methodological limitations insofar as feedback was restricted to the information that was contained in the allocated task card, thereby restricting the utility of these findings. To this point, there has been no research on the effectiveness of skill development, or physical activity and PE-related behavior measures in which a non-reciprocal, formalized (i.e., training of peer-teachers who were provided with a teaching resource package – termed ‘T-PT’), small group peer teaching approach was used within an intact secondary school PE class setting. In order to overcome these limitations, peer-teachers in this study were trained using a purposefully designed resource booklet specific to the tactical games approach (Griffin et al., 1997) to structure the lessons delivered by the peer-teachers.

The tactical games approach emphasizes game play and attempts to place the learning of motor skills within the relevant game context. The primary goal of this approach is to improve students’ game performance. Under the tenets of the tactical games approach, lessons begin with a developmentally appropriate game, followed by discrete skill practice tasks that lead to the reapplication of these skills in another game. Each lesson is presented in a tactical framework with a game-questions-practice-game sequence, which serves to reinforce game goals, and develop tactical awareness and skills (Griffin et al., 1997). Advocates of a games approach believe it to be highly motivating and that it allows for meaningful play (Mitchell et al., 2003), and this approach is considered to be an important PE pedagogy (Thorpe et al., 1984; Griffin et al., 1997), thereby making it a worthy strategy to underpin the soccer lessons in this research.

In summary, in order to address the paucities in the existing literature, this study was based on non-reciprocal, small group peer teaching within an intact secondary school PE class setting. The aim of this study was to determine the effects of formalized peer-teacher training (intervention) on peers’ learning outcomes to include soccer performance, engagement level of in-class physical activity, perceived enjoyment, learning climate, structured learning time, and ALT-PE, as well as to determine the influence of the intervention on the peer-teachers’ quantity of feedback, enjoyment and motivation. It was also hypothesized that the students who received instruction under the conditions of T-PT would display higher-quality teaching practices than untrained peer-teachers, as evidenced via their provision of greater ALT-PE, feedback, and structured learning activities than those taught by untrained peers (U-PT). We also forecasted that trained peer-teachers (T-PT), at the conclusion of the intervention, would report higher PE motivation and enjoyment outcomes than untrained peer-teachers (U-PT). For those students who were working under T-PT (as opposed to U-PT), we hypothesized that they would display higher PA levels, greater PE enjoyment, and more positive learning climate perceptions. Finally, we anticipated that students receiving instruction from trained peers (T-PT) would display greater motor skill (i.e., soccer performance) improvement from pre- to post-intervention than those students taught by untrained peers (U-PT).

## MATERIALS AND METHODS

### PARTICIPANTS

The sample comprised eight intact year 7 and 8 PE classes (*n* = 200) in a co-education metropolitan secondary school in Perth, WA, Australia. The participants, 106 males and 94 female were between the ages of 11 and 13 (*M* = 12.46; *SD* = 0.59). Four of the eight classes were randomly selected to form the intervention group (*n* = 94; T-PT) and four classes to form the control group (*n* = 106; U-PT). In each class, either three or four students volunteered to serve as peer-teachers for the small group instruction (resulting in 17 males, 15 females in total). The exact number of peer-teachers in each class was determined by class size, in order to ensure that each peer-teacher was responsible for a group of ∼4 ‘student recipients’ (*n* = 168). Teachers only endorsed students to serve as peer-teachers if they fulfilled the criteria identified by Houston-Wilson et al. (1997): (a) in the same class as their classmates, (b) proficient in relevant sports skills, (c) good behavior in PE classes, and (d) a strong desire to be a peer-teacher in the study. In addition, the class PE teacher also considered the students’ compliance and ability to engage their peers. Teachers reported that no volunteer students failed to meet the criteria and were therefore all accepted. A sub-sample of 16 student recipients from the T-PT group and 16 from the U-PT group undertook a pre- and post-unit soccer performance test. These students were determined to have regular PE attendance and possess a range of soccer abilities as determined by the school’s Head of PE (HOD). These 32 students also wore an ActiGraph GT1M accelerometer to measure in-class physical activity levels during each lesson in the study.

### INTERVENTION DESIGN AND PROCEDURES

After obtaining permission to conduct the study from the Human Research Ethics Committee at the authors’ institution, permission for participant involvement was sought from the principal, teachers, parents/guardians, and students through a set of documents detailing research aims and procedures, statements of disclosure, and details of the researchers. Passive consent forms were provided to parents/guardians to enable them to withdraw their child/ward’s involvement. Students and their parents were blinded to the true purpose of the study, and members of T-PT and U-PT groups were not informed about the teaching practices that were being used in the other condition. All students were fully debriefed and provided with all training materials following the completion of the investigation. All PE lessons took place on an outdoor grassed sports field. All students were provided with appropriate sized soccer balls, as determined by the HOD. Colored plastic field domes were also provided in both conditions in order to demarcate the soccer fields and goals. All pre- and post-intervention performance testing was undertaken in an indoor sports hall, using the full-sized basketball court markings (28.6 m × 15.2 m) to demarcate the soccer field, and colored plastic field domes to identify the goal area.

Variables of interest were measured with peer-teachers and student recipients prior to, during, and following a 5-week PE soccer unit. During the study period, the peer-teachers from both groups were asked to teach for 30-min blocks during a series of 10 PE lessons. The 10 target lessons, which were each 55 minutes in total duration, were spread evenly over a 5-week period. Peer-teachers were asked to access a sequence of standardized soccer concepts and pedagogy that were consistent with a tactical games approach detailed in Griffin et al. (1997). The proposed soccer curriculum was presented to, and validated for suitability by, the school’s HOD, a teacher of 10 years’ experience, prior to the start of the program. The curriculum included: (a) maintaining possession of the ball, (b) attacking the goal, (c) defending space, (d) defending the goal, and (e) restarting play from a throw in and corner kick. At the beginning of the program, peer-teachers in the T-PT group attended three 20-min teaching-focused training sessions delivered by the HOD and were provided with an instructional manual containing lesson objectives, an equipment list, teaching activities, class questions, skill-related learning cues, and self-assessment learning checklists.

In the first training T-PT session, students were provided with a copy of the teaching resource, given an opportunity to familiarize themselves with its contents and introduced to the tactical games approach pedagogy. With the HOD teaching, and the students participating in a micro-lesson focusing on the inside foot pass and soccer ball trap, students were introduced to eight qualities of PE teaching (Soh, unpublished master’s thesis); including, (a) good student and teacher positioning, (b) giving a brief lesson introduction, (c) providing an accurate demonstration with teaching cues, (d) using clear explanation and clarification techniques, (e) providing appropriate practice, (f) using focused observation and analysis, (g) providing positive feedback (including action, general, specific, and corrective feedback), and (h) suitable task modifications for enjoyment and learning. The second training session involved the HOD revising the 10 tactical games approach lessons and delivering the first prescribed lesson, ‘passing and receiving balls on the ground with the inside of foot’ with the T-PT students participating. The HOD reinforced the tactical games approach sequence of events, including game-questions-practice-game, whilst revising the qualities of teaching PE content. The third training session required the T-PT students to divide into two groups and using the prescribed practice game from lesson 10 (attacking at corner kicks), students rotated through the teachers role, involving practice related to monitoring student performance, giving feedback to players and reinforcing teaching cues. At the conclusion, students were given an opportunity to clarify the peer-teaching expectations with the HOD, aspects related to the tactical games approach, and reminded to revise their instructional resource before coming to each peer-teaching class. Peer-teachers within the U-PT group were also required to provide lesson activities consistent with these concept headings; however, they did not receive training or the instructional resource manual, and were not instructed as to the specific activities or pedagogy to employ. Throughout the 10 lessons of the study, the class PE teacher was instructed to only intervene if s/he believed that the safety of students was compromised (no requirement for teacher intervention was reported).

#### Teaching verification

All of the lessons taught by the T-PTs were observed by two researchers and video recorded using two digital video recorders, one situated perpendicular to a sideline and the other behind a goal line. In addition, the peer-teachers had a digital voice recorder strapped to the peer-teacher’s bicep. During and after each lesson, the audio and video recordings were analyzed by the two field researchers to verify appropriate implementation of the prescribed teaching activities and pedagogies as detailed in the training manual. No inconsistencies were identified.

### MEASURES

Self-report data were collected in relation to PE enjoyment (for both recipients and peer-teachers), motivation (peer-teachers only), and perceptions of autonomy support (recipients only). Measures were also obtained of in-class physical activity, type of peer-teacher feedback, ALT-PE, soccer performance, and structured learning time. Focus-group interviews were conducted at the end of the study to examine the general perceptions of the peer-teachers about the teaching and learning experience.

#### Student recipient outcome measures

***PE enjoyment*.** Enjoyment was measured pre- and post-treatment using four items from the interest/enjoyment subscale of the Intrinsic Motivation Inventory (IMI; Ryan, 1982). The generic label of “activity” was replaced with “PE lessons” in order to ensure all items were suited to the context under investigation. Students responded to the items using a seven-point response format ranging from 1 (*not at all true*) to 7 (*very true*), and items included “I enjoy my PE lessons very much,” and “my PE lessons are fun to do.” In line with existing work (e.g., Dimmock et al., 2013), three items from the original subscale that explicitly measure interest were excluded from the instrument used in this investigation. The enjoyment subscale of the IMI has been widely used in previous PE-based investigations (e.g., Jackson et al., 2012), and measures produced from this instrument have consistently demonstrated adequate factor structure and internal reliability, as well as favorable correlations with theoretically related variables (e.g., effort, perceived competence; Goudas et al., 1995; Koka and Hein, 2003). Alpha coefficients for pre- and post-treatment enjoyment measures among student recipients were 0.91 and 0.92, respectively.

***Perceptions of autonomy support*.** The Learning Climate Questionnaire (LCQ; six-item abridged version), developed by Williams and Deci (1996), was administered pre- and post-study such that the student recipients’ perceptions of the PE lessons could be examined in terms of autonomy support. This enabled comparisons between their regular PE and peer-based PE experiences. In this study, the six-item version of the LCQ was used, with the word “instructor” changed to “teacher” for all items (How et al., 2013). Example items include, “I feel that my teacher provides me choices and options,” and “My teacher tries to understand how I see things before suggesting a new way to do things.” Responses were measured on a seven point scale, anchored at 1 (*strongly disagree*) and 7 (*strongly agree*). Overall scores were calculated by averaging responses across the six items, whereby higher average scores represent a higher level of perceived autonomy support. Alpha coefficients for pre- and post-treatment learning climate measures among student recipients were 0.87 and 0.93, respectively.

***In-class physical activity*.** Sixteen students in each condition (T-PT and U-PT; 16 males and 16 females) were selected to wear ActiGraph GT1M accelerometers to determine physical activity levels during the peer teaching sessions. These students were the same cohort selected to undertake the pre- and post-intervention soccer performance test and were defined by the HOD to have regular PE attendance and possess a range of soccer abilities. A 15-sec interval length was used to capture the raw counts of physical activity intensity and volume in each peer teaching session (Trost et al., 2002, 2005; Freedson et al., 2005). The percentage of time spent in physical activity was calculated using Trost et al. (2002) count thresholds: (a) light intensity: <598; (b) moderate intensity: between 599 to 1343; and (c) vigorous intensity: >1344. The percentage of time spent within a specific intensity was determined by summing all counts within a count threshold (Freedson et al., 2005).

***Soccer performance*.** Thirty-two students (16 U-PT group participants and 16 T-PT group participants) participated in a pre- and post-intervention soccer performance evaluation. Specifically, the Games Performance Assessment Instrument (GPAI) developed by Oslin et al. (1998) was used to measure soccer performance. The assessments involved four different soccer games using a 28.6 m × 15.2 m field. Small sided games (four per side) in a relatively large indoor space restricted the possibility for concerns relating to not registering an appropriate or efficient performance, or accruing a relatively similar number of performance actions across participants (Memmert and Harvey, 2008). These students were selected by the HOD to represent a range of abilities (i.e., to include relatively good, moderate, and weak players). An approximately equal number of boys and girls were selected in each team. These soccer games consisted of two 10-min halves, with four students per team and a 1-min rest interval. The games were captured using two Sony SR42E digital video recorders. Pre- and post-intervention student performance was coded independently by two experienced soccer coaches who undertook pre-training as recommended by Memmert and Harvey (2008). Three components of GPAI were coded: (a) decision making, making appropriate choices about what to do with the ball during a game, (b) skill execution, efficient performance of the skills taught, and (c) support, off-the-ball movement to a position to receive a pass when player’s team has possession. Consistent with previous recommendations (Oslin et al., 1998), the criteria for assessing game performance were created from the content taught in the classes and at a level of expectation, as confirmed by the HOD, for students who are effective performers of the defined skills in the PE class context. As recommended, relatively narrow and specific criteria were defined (Memmert and Harvey, 2008). Example criteria for assessing ‘decision making’ included holding the ball up when appropriate to allow teammates to get free, shooting at goal when appropriate, not stopping with the ball when appropriate, and allowing defenders to ambush the ball carrier. Example criteria for assessing ‘skill execution’ for passing included facing the direction of the passing target, having the ball reach the passing target, and for throw-ins, throwing the ball to the feet of the receiver and move into play. Example criteria for the assessing ‘support’ included creating a short passing option, moving in behind the marker to receive a pass, and positioning to score from the first touch of a corner kick. These individually observable behaviors were assessed in terms of whether they were efficient/inefficient (skill execution), and appropriate/inappropriate (decision making and support; Oslin et al., 1998). To avoid previous concerns associated with index calculations (Memmert and Harvey, 2008), a simple dichotomous scoring system was employed (i.e., efficient/inefficient and appropriate/inappropriate) and these values were used for analysis at pre- and post-intervention. Inter-observer agreement (IOA) for the two coders was calculated using the formula: $IOA=\frac{agreements}{\left(agreements+disagreements\right)}*100$ (van der Mars, 1989; Thomas et al., 2005). The IOA percentage score was found to be 90.5 for the two coders.

***Active learning time – physical education (ALT-PE)*.** The ALT-PE instrument (Siedentop et al., 1979) was used to record the following representative behaviors: (a) motor appropriate, (b) motor inappropriate, (c) motor supporting, and (d) not motor engaged, which was further categorized as follows: interim, waiting, off-task, on-task, and cognitive. Each peer teaching cluster (four per class) from each of the eight classes was video-taped during the 5-week intervention. A total of 32 peer teaching video clips (16 U-PT clusters and 16 T-PT clusters) were captured and analyzed independently by a project assistant (an experienced PE teacher) and the first author who were blinded to the assignment of peer-teachers (U-PT or T-PT). The analysis was performed on three randomly selected target students in each cluster as well as the whole group. Using the timer in the Windows Media Player, each target student was observed for a period of 10 sec every 2 min. The whole group was observed at the end of every fourth minute. Whole group observation involved scanning all students once only from left to right of the screen. An average of 15 observations per session was recorded for each target subject and for the whole group. A sum and a percentage score were obtained for each representative behavior, and a mean score and a mean percentage score were calculated for the observed session. Observations stopped during break times and when the class teacher intervened within the target peer teaching cluster. Therefore, the number of observations for each target student and the whole group differed between sessions depending on the actual time available for peer teaching, as well as the length of breaks. The IOA between the two researchers was 82.14%.

#### Pedagogical outcome measures

The quality of peer teaching was analyzed by two experienced pedagogical researchers in line with recommendations by Rink (2002). Moreover, pedagogical outcomes were also determined by amount of class time engaged in structured versus unstructured lesson activities.

***Quantity of peer-teacher feedback*.** A sample of two-or-three 30-min peer teaching sessions for each peer-teacher was captured with an Olympus WS-110 digital voice recorder (*n* = 40 recordings per condition). A total of 80 peer teaching audio clips were transcribed verbatim by the researcher to determine the quantity of feedback provided by peer-teachers to students. Transcripts were analyzed independently by a project assistant (an experienced PE teacher) and the first author who were blinded to the group of these peer-teachers (U-PT or T-PT). A tally system was used to record the following categories of feedback (Rink, 2002): (a) target – group or individual; (b) type – evaluative or corrective; (c) level of specificity – general or specific; and (d) positive or negative. A sum and a percentage score were obtained for each representative feedback, and a mean score and a mean percentage score were calculated for the observed session. The IOA between the two researchers was 80.15%.

***Structured/unstructured learning time*.** Consistent with the concept of deliberate practice (Starkes and Ericsson, 2003), the amount of class time that the peer-teachers allocated to structured and unstructured activities was assessed. Structured or learning focused activities were those that aligned with the lesson outcomes and were closely monitored while unstructured or non-learning focused activities were those that did not align with the learning outcomes and were loosely monitored (Cote et al., 2003). Moreover, structured learning activities were defined as those that were front-loaded by goal-focused instructions, including demonstrations, within task instructions, demonstrations and augmented feedback that related to the lesson learning outcomes. Unstructured lesson time was determined when lesson activities occurred in the absence of any of the criteria that defined structured learning activities. Therefore, unstructured lesson time included, for example, a game of soccer where no skill or strategic focus was identified in the pre-game instructions or reinforced through the game and post-game feedback; that is, where ‘fun’ rather than learning was the focus of the activity (Starkes and Ericsson, 2003). Each peer teaching cluster from each of the classes was video-taped on one occasion during the 5-week intervention. Consequently, a total of 32 peer teaching video clips (16 U-PT clusters and 16 T-PT clusters) were captured and analyzed independently by the researcher and the first author, and the number of minutes allocated to structured and unstructured lesson activities was recorded. The IOA between the two researchers was 92.52%.

#### Peer-teacher outcome measures

***PE enjoyment*.** Enjoyment perceptions of all students, including the peer-teachers was measured pre- and post-study using four items from the IMI (Ryan, 1982) interest/enjoyment subscale. Alpha coefficients for pre- and post-treatment learning climate measures among peer-teachers were 0.86 and 0.84, respectively.

***PE motivation*.** Peer-teacher motivation in PE was measured using an instrument that was previously adapted for high school PE research (SMSPE; Ward et al., 2008; How et al., 2013) from the sport motivation scale (SMS; Briere et al., 1995; Pelletier et al., 1995). The SMS is a valid and reliable tool for measuring motivation (Pelletier et al., 1995). Modifications to the original SMS included changing the word “sport(s)” to “PE,” in order to ensure contextual relevance while maintaining the original meaning of the items. The SMSPE measured students’ contextual motivational orientations toward PE, and consisted of 28 statements split evenly across seven domains. The seven domains within the SMSPE include: (a) intrinsic motivation (IM), consisting of IM accomplishments, IM stimulation, and IM to know, (b) extrinsic motivation (EM) identified, (c) EM introjected, (d) EM external regulation, and (e) amotivation. The three IM components were combined and averaged to provide a mean IM score. Student responses were made on a 7-point Likert-like scale ranging from 1 (*does not describe me at all*) to 7 (*describes me exactly*). Alpha coefficients observed for all subscales at pre- and post-treatment in this investigation were as follows: IM accomplishments (α = 0.81 and 0.90), IM stimulation (α = 0.71 and 0.85), IM to know (α = 0.66 and 0.83), identified regulation (α = 0.74 and 0.87), introjected regulation (α = 0.77 and 0.77), external regulation (α = 0.79 and 0.81), and amotivation (α = 0.68 and 0.68). A self-determination index (SDI), derived from students’ scores on the various SMSPE subscales, was calculated to identify the extent to which students’ actions were self-determined, or autonomous (relative to more controlled forms of motivation). To calculate the SDI, weightings were used in line with previous guidelines (IM to know, +2; IM accomplishments, +2; IM stimulation, +2; EM identified, +1; EM introjected, -1; EM external regulation, -1; amotivation, -2; Vallerand, 2001).

***Post-study interviews with peer-teachers*.** Two small focus group semi-structured interviews were conducted by a trained project assistant (an experienced PE teacher), one with six peer-teachers from the U-PT group, and one with six peer-teachers from the T-PT group. A semi-structured interview guide was initially piloted with three faculty members who were experienced in conducting semi-structured interviews and were familiar with the pedagogical literature. The pilot process assisted in developing the breadth and depth of the interview guide, ensuring the appropriateness of questions, and enabling the identification of problematic phrases or wording. The final semi-structured interview guide comprised of questions specific to positive outcomes and challenges of their peer-teaching experience (see **Table 1**). The interview guide for U-PT and T-PT groups focused on specific, pre-determined issues, but at the same time permitted flexibility and additional probing from the researcher (Whittemore et al., 2001).

**Table 1:** Post-intervention perceptions of peer teaching of the trained and untrained peer teachers.

		Frequency of comments from T-PT/U-PT					
Category		Theme	T-PT	(%)	U-PT	(%)	Total
Positive outcomes
		Improved learning	16	72.7	6	27.3	22
		Enjoyment	15	68.2	7	31.8	22
		Good experience	15	78.9	4	21.1	19
		Self-esteem/confidence	11	61.1	7	38.9	18
		Found the instructional manual useful	19	100.0	NA	–	19
		Training useful	7	100.0	NA	–	7
		Self-assessment checklist useful	10	100.0	NA	–	10
		Total (positive outcomes)	93		24		117
Challenges
		Misbehavior	5	50	5	50	10
		Lack of respect	3	27	8	73	11
		Lack knowledge/ideas	4	36	7	64	11
		Group too small	1	25	3	75	4
		Lack of support/training	0	0	9	100	9
		Self-assessment checklist not useful	1	100	NA	–	1
		Others	2	50	2	50	4
		Total (challenges)	16		34		50

*T-PT, Formalized (i.e., trained and resource supported) Non-Reciprocal Peer Teachers. Numbers represent the frequency of comments provided within a given theme by T-PT/U-PT. For example, in the Positive Outcomes category, there were 16 separate comments from T-PT program teachers about improved student learning, in comparison to six comments regarding this theme from U-PT*.

Each interview lasted ∼20 min. Focus group interviews of six students were chosen in line with recommendations for participants who are similar to and cooperative with each other (Creswell, 2008). The peer-teachers who were interviewed were identified by the HOD to have met the study criteria. These focus group sessions were designed to gather further information regarding the peer-teachers’ perceptions of the peer-teaching experience. Six questions were asked: (a) Tell me about your peer teaching experience; (b) How prepared were you for each lesson that you taught?; (c) What was the best thing that happened to you as a peer-teacher?; (d) What was the most frustrating thing about peer teaching?; (e) Please describe how much you enjoyed the peer teaching; and (f) Do you think you should have some form of training before you start peer teaching? The group interviews were recorded and transcribed for purpose of analysis. To maintain the anonymity of the peer-teachers, no student or peer-teacher names were included in the transcriptions of the group interviews.

### DATA ANALYSIS

All quantitative data were analyzed using SPSS, and prior to running primary analyses, data screening checks were performed and descriptive statistics computed. For student recipient and peer-teacher data, a series of ANOVAs and MANOVAs were computed to examine differences on the dependent variables (i.e., soccer performance; perceptions of enjoyment, autonomy support, and motivation; intensity of physical activity; and behavior of teaching) according to time (where relevant) and experimental conditions.

Post-intervention interview transcripts were deductively analyzed for themes through open and selective coding (Burns, 2000). Open coding was used to select, identify, and label categories as part of the data analysis. Subsequently, selective coding validated the initial conclusions and further refined the themes relating to teaching preparation, and for perceptions pertaining to the peer-teaching experience, positives, negatives and enjoyment. Two experienced researchers cross-validated the lead author’s analysis by reading and independently coding a sample of meaning units. Specifically, the first author presented themes and meaning units to researchers for comparisons to be made with the original researcher’s coding, and any areas of disagreement were resolved through re-analysis and group discussion. This process ensured that all meaning units were grouped under appropriate themes, and allowed for consensus between researchers regarding the meaning of participants’ responses.

## RESULTS

Physical education enjoyment (recipients and peer-teachers), autonomy support (recipients only), and motivation (peer-teachers only) are compared for the U-PT and T-PT groups for time one (representing teacher-centered PE) and time two (post peer teaching).

### STUDENT RECIPIENT OUTCOMES

#### PE enjoyment

A 2 (time) × 2 (condition) mixed-model ANOVA was used to test for differences in the recipients’ perceptions of enjoyment. Analyses revealed no significant main effects for condition (*F*_1,159_ = 0.05, *p* = 0.83, $\eta_{p}^{2}$ < 0.001) or time (*F*_1,159_ = 0.10, *p* = 0.75, $\eta_{p}^{2}$ = 0.001), as well as no significant condition-by-time interaction effect (*F*_1,159_ = 0.77, *p* = 0.38, $\eta_{p}^{2}$ = 0.005). These analyses indicated no between group changes in the student recipients’ pre- and post-study scores for the level of enjoyment in PE.

#### Autonomy support

A 2 (time) × 2 (conditions) mixed-model ANOVA was again used to examine differences in the recipients’ perceptions of autonomy support (i.e., using LCQ scores). Analyses revealed no significant main effects for condition (*F*_1,160_ = 0.02, *p* = 0.88, $\eta_{p}^{2}$ < 0.001) or time (*F*_1,159_ = 0.72, *p* = 0.40, $\eta_{p}^{2}$ = 0.004), as well as no significant condition-by-time interaction effect (*F*_1,159_ = 0.52, *p* = 0.47, $\eta_{p}^{2}$ = 0.003). These analyses indicated no between group changes were apparent in the student recipients’ pre- and post-study scores for autonomy support.

#### In-class physical activity

Using data collected from the sub-sample of student recipients who wore accelerometers, a one-way MANOVA was conducted to explore potential differences in activity levels between groups during the intervention. The percentage of class time spent in light, moderate, and vigorous physical activity, were entered as dependent variables, and univariate significance levels were adjusted accordingly (i.e., α = 0.0166). No significant multivariate effect was revealed (*F*_3,28_ = 0.87, *p* = 0.47, $\eta_{p}^{2}$ = 0.08, λ = 0.92). There were no between group differences for time spent in light, moderate, or vigorous physical activity (significance values at the univariate level ranged between 0.32 and 0.91).

#### Soccer performance

Two performance indices were calculated; the first reflected the total number of efficient/appropriate performance behaviors engaged in by game participants, and the second reflected the number of inefficient/inappropriate in-game performance behaviors. Although 16 participants in each condition were targeted for video-based game performance assessment, footage clarity/dropout and field capture limitations resulted in complete pre- and post-performance assessments being obtained from 11 participants in each condition. A 2 (time) × 2 (condition) mixed-model ANOVA was first used to assess differences on participants’ efficient/appropriate performance actions. This ANOVA yielded no significant main effect for condition (*F*_1,20_ = 0.14, *p* = 0.72, $\eta_{p}^{2}$ = 0.007), alongside a significant main effect for time (*F*_1,20_ = 6.50, *p* = 0.02, $\eta_{p}^{2}$ = 0.24). This main time effect, however, was superseded by a significant condition-by-time interaction effect that was large in magnitude (*F*_1,20_ = 13.60, *p* = 0.001, $\eta_{p}^{2}$ = 0.40). Follow-up analyses of the interaction effect were computed using paired-sample *t*-tests to examine change-over-time for each condition separately. Analyses demonstrated that there were no significant differences between time 1 (*M* = 20.36, *SD* = 9.53) and time 2 (*M* = 17.81, *SD* = 9.02) regarding the number of efficient/appropriate in-game actions performed by participants in the U-PT group (*t*_10_ = 0.74, *p* = 0.48). However, participants who had been instructed by trained peer-teachers (T-PT) displayed a significant improvement in terms of efficient/appropriate behaviors from time 1 (*M* = 13.45, *SD* = 10.11) to time 2 (*M* = 27.41, *SD* = 11.21; *t*_10_ = -4.89, *p* = 0.001).

With respect to inefficient/inappropriate in-game performance, a separate 2 (time) × 2 (conditions) mixed-model ANOVA revealed a significant main effect for time (*F*_1,20_ = 7.04, *p* = 0.02, $\eta_{p}^{2}$ = 0.26), alongside no significant main effect for condition (*F*_1,20_ = 0.01, *p* = 0.95, $\eta_{p}^{2}$ < 0.001), and no significant condition-by-time interaction effect (*F*_1,20_ = 0.01, *p* = 0.97, $\eta_{p}^{2}$ < 0.001). The significant time effect indicated that, irrespective of whether participants had received instruction from T-PT or U-PT, there was a significant increase in the number of inefficient/inappropriate actions observed between time 1 (*M* = 8.70, *SD* = 4.96) and time 2 (*M* = 13.32, *SD* = 6.23).

#### ALT-PE

In order to test for potential differences on global representative behaviors, we performed a MANOVA to examine scores for targeted U-PT and T-PT intervention students on motor appropriate, motor inappropriate, and motor supporting categories (with an adjusted univariate α = 0.017 to account for multiple comparisons). Analyses revealed a significant multivariate effect (*F*_3,20_ = 5.72, *p* = 0.005, $\eta_{p}^{2}$ = 0.46, λ = 0.54), which was accounted for by differences on motor appropriate (*F*_1,22_ = 10.40, *p* = 0.004, $\eta_{p}^{2}$ = 0.32) and motor inappropriate (*F*_1,22_ = 7.14, *p* = 0.014, $\eta_{p}^{2}$ = 0.24), but not motor supporting (*F*_1,22_ = 1.88, *p* = 0.18, $\eta_{p}^{2}$ = 0.08) activities. In particular, those who were instructed by T-PT (*M* = 18.08, *SD* = 5.63) displayed greater motor appropriate behavior than those with the U-PT (*M* = 11.42, *SD* = 4.42), as well as lower motor inappropriate activity (*M*_trained_ = 0.00, *SD*_trained_ = 0.00; *M*_untrained_ = 2.33, *SD*_untrained_ = 3.03).

A second MANOVA was subsequently performed to examine whether students within the U-PT and T-PT intervention conditions differed according to the five separate ‘not motor engaged’ (i.e., interim, waiting, on-task, off-task, cognitive) activities, using an adjusted univariate significance level (α = 0.01). A significant multivariate effect emerged (*F*_5,18_ = 3.44, *p* = 0.02, $\eta_{p}^{2}$ = 0.49, λ = 0.51), which was accounted for solely by differences on on-task activities (*F*_1,22_ = 9.12, *p* = 0.006, $\eta_{p}^{2}$ = 0.29). Follow-up analyses indicated that students working under T-PT (*M* = 12.08, *SD* = 5.33) displayed greater on-task behaviors than those under untrained peer-teachers (*M* = 5.58, *SD* = 5.21) when not motor engaged.

### PEDAGOGICAL OUTCOMES

The researchers’ observations confirmed that T-PT lessons were delivered in accordance with the specified tactical games approach identified in the manual, with a game-questions-practice-game sequence. Whilst the same tactical concepts were observed to be the focus of the U-PT lessons (as requested by the researchers), command style and practice style were exclusively used to deliver soccer skills and no tactical games approach or resource-prescribed games were used.

#### Type of peer-teacher feedback

In order to determine whether differences existed for feedback between U-PT and T-PT, a series of four MANOVAs were computed, each comprising two dependent variables that reflected separate conceptual components of feedback. Specifically, separate MANOVAs were performed for variables representing (a) *target* of feedback (i.e., group and individual), (b) *type* of feedback (i.e., evaluative and corrective), (c) *specificity* of feedback (i.e., general and specific), and (d) *tone* of feedback (i.e., positive and negative), with univariate significance levels adjusted accordingly (i.e., α = 0.025). The MANOVA for feedback target revealed no significant multivariate effect (*F*_2,29_ = 2.06, *p* = 0.15, $\eta_{p}^{2}$ = 0.12, λ = 0.88), as did the MANOVA for feedback type (*F*_2,29_ = 1.38, *p* = 0.27, $\eta_{p}^{2}$ = 0.09, λ = 0.91), indicating that U-PT and T-PT did not differ in their provision of group, individual, evaluative, or corrective feedback.

For specificity of feedback, a significant multivariate effect emerged (*F*_2,29_ = 6.26, *p* = 0.005, $\eta_{p}^{2}$ = 0.30, λ = 0.70), which was accounted for by differences in the amount of general (*F*_1,30_ = 11.32, *p* = 0.002, $\eta_{p}^{2}$ = 0.27) and specific (*F*_1,30_ = 1.69, *p* = 0.20, $\eta_{p}^{2}$ = 0.05) feedback provided. That is, T-PT (*M* = 5.20, *SD* = 2.09) provided greater general feedback than U-PT (*M* = 3.28, *SD* = 0.39). Finally, for tone of feedback, there was again a significant multivariate effect (*F*_2,29_ = 12.58, *p* < 0.001, $\eta_{p}^{2}$ = 0.46, λ = 0.54), which in this case was accounted for by differences on both positive (*F*_1,30_ = 6.57, *p* = 0.02, $\eta_{p}^{2}$ = 0.18) and negative (*F*_1,30_ = 10.42, *p* = 0.003, $\eta_{p}^{2}$ = 0.26) feedback. In particular, it emerged that T-PT provided more positive feedback (*M*_trained_ = 18.13, *SD*_trained_ = 6.32; *M*_untrained_ = 12.80, *SD*_untrained_ = 5.43) and more negative feedback (*M*_trained_ = 1.79, *SD*_trained_ = 0.84; *M*_untrained_ = 1.03, *SD*_untrained_ = 0.40) than their U-PT counterparts.

#### Structured/unstructured learning time

A MANOVA was conducted to explore potential differences in terms of the minutes of structured learning time provided by U-PT versus T-PT intervention classes. A significant effect emerged for condition (*F*_1,64_ = 6.52, *p* = 0.013, $\eta_{p}^{2}$ = 0.09), insofar as T-PT, on average, provided a significantly greater amount of structured learning time (*M* = 23.05 min, *SD* = 5.07) when compared to their U-PT counterparts (*M* = 18.87 min, *SD* = 7.72).

### PEER-TEACHER OUTCOMES

#### PE enjoyment

A 2 (time) × 2 (condition) mixed-model ANOVA revealed no significant main effects for condition (*F*_1,30_ = 0.26, *p* = 0.62, $\eta_{p}^{2}$ = 0.008) and no significant condition-by-time interaction effect (*F*_1,30_ = 0.20, *p* = 0.66, $\eta_{p}^{2}$ = 0.007). However, a significant time effect was apparent (*F*_1,30_ = 8.30, *p* = .0007, $\eta_{p}^{2}$ = 0.22), insofar as peer-teachers (irrespective of whether they were in the U-PT or T-PT condition) reported greater enjoyment of PE at post- (*M* = 6.19, *SD* = 0.79) compared to pre-study (*M* = 5.91, *SD* = 0.70).

#### PE motivation

A 2 (time) × 2 (condition) mixed-model ANOVA was used to examine potential differences on teachers’ SDI scores (calculated from SMSPE responses). No significant main effects emerged for condition (*F*_1,30_ = 1.17, *p* = 0.29, $\eta_{p}^{2}$ = 0.04) or time (*F*_1,30_ < 0.001, *p* = 0.99, $\eta_{p}^{2}$ < 0.001), and no significant condition-by-time interaction effect was observed (*F*_1,30_ = 2.46, *p* = 0.13, $\eta_{p}^{2}$ = 0.08). These analyses indicated that no changes were apparent between baseline (i.e., pre-study) and peer-based (i.e., post-study) levels of motivation for T-PT and U-PT.

#### Interviews with peer-teachers

All interview data were analyzed with the purpose of creating common themes relating to any benefits and/or noteworthy aspects of the peer teaching (Burns, 2000). A summary of the number of comments provided by T-PT and U-PT within each of the emergent themes is provided in **Table 1**, and meaning units (i.e., comments) from both cohorts are presented in the discussion section in order to assist with interpretation of results.

### SUMMARY OF RESULTS

Taken together, the lack of interaction effects that were observed for self-report variables (i.e., enjoyment, autonomy support, motivation) revealed no evidence of differing patterns of change over time for student recipients and/or peer-teachers. We also observed that students within the U-PT and T-PT conditions appeared to engage in relatively similar patterns of in-class physical activity behavior. We did, however, observe a number of desirable effects associated with the T-PT condition when considering behavioral and pedagogical outcomes. First, student recipients working under T-PT displayed a significant improvement in their efficient/appropriate in-game performance actions over the course of the intervention (which was not apparent for those under U-PT). Interestingly, irrespective of whether participants had received instruction from a trained or untrained peer-teacher, we also witnessed a significant increase in the number of inefficient/inappropriate actions observed at post- (relative to pre-) treatment. Second, relative to those in the U-PT condition, students who worked within a tactical games framework whilst receiving instruction from T-PT displayed increased motor appropriate behavior, lower motor inappropriate behavior, and greater on-task behavior when not motor engaged. Third, T-PTs (relative to U-PT) were shown to display greater general feedback, positive feedback, and negative feedback during their periods of instruction. Fourth, we observed that the T-PT working within a prescribed tactical games approach provided a significantly greater amount of structured learning time than those who did not receive training and chose the pedagogy. Finally, our qualitative analyses revealed that T-PT reported a range of more positive feelings about peer teaching and the intervention program, along with fewer perceived challenges associated with accessing peer teaching and learning outcomes, when compared to those who did not receive the formal training.

## DISCUSSION

Although PE teaching effectiveness has been correlated with student compliance, issues relating to lesson differentiation, quality pedagogy, and student learning require further investigation. Peer teaching has the potential to influence student outcomes (Siedentop and Tannehill, 2000; Whipp et al., 2012); however, a large proportion of existing peer teaching studies have been conducted in special educational and/or primary school settings, have used bi-directional (i.e., reciprocal) teaching, and have not provided formal teaching support for the peer instructor. To address these gaps in the literature, the effectiveness of delivering a peer-teacher training program to high school students was examined in terms of student/peer-teacher psychosocial behaviors, pedagogical behaviors and student learning.

Based on previous work (Carlson and Hastie, 1997; Cai, 1998; Cecchini et al., 2001; Cothran and Kulinna, 2006), it might be considered surprising that student recipient and peer-teacher levels of PE enjoyment, autonomy support (student recipient only), and motivation (peer-teachers only) did not differ between the U-PT and T-PT groups across time one (representing teacher-centered PE) and time two (post peer teaching). While the relative differences for the formalized (T-PT intervention) and non-formalized (U-PT) classes may not have resulted in perceived variation for the students, the lack of differences between the teacher-centered pedagogy (time one) and peer teaching program (time two) is consistent with existing research (Iserbyt et al., 2011). Although we observed no statistical effects on these indices, the T-PT did provide significantly more comments (see **Table 1**) regarding the enjoyment arising from their teaching experiences during the post-program interview: “…like when you’re getting in and doing it with them, it’s like much more fun rather than the teacher doing it” (T-PT); and, “It was really fun to get out there to see how it is to be like on the other side of the sport lesson” (T-PT). Perceptions of autonomy were also highlighted during the interviews: “It was fun teaching because you can become the boss” (U-PT); and, “The teacher has so much control, like it gives you this power… it’s a good feeling” (T-PT). Finally, comments about motivating students were also apparent among the T-PT peer-teachers: “I sort of tried to get them motivated with the activities that were in the [training] book” (T-PT). In contrast, some untrained peer-teachers were less positive: “When I first thought about it, I thought it was going to be better but when it turned out, because people have attitudes” (U-PT); and “It was difficult... because you don’t have the same authority as a teacher, so... that’s the one thing... that’s the main point” (U-PT).

Students working under U-PT and T-PT displayed similar in-class physical activity behavior; however, those working under T-PT did display significantly greater motor appropriate behavior, lower motor inappropriate behavior, and greater on-task behavior when not motor engaged. While this is an encouraging finding, and is consistent with some existing peer-teacher research (dePaepe, 1985; Iserbyt et al., 2011), it is unknown if the ALT-PE recorded in this study would be equivalent to (or might even exceed) that generated when using a teacher-centered approach (Iserbyt et al., 2011). Further research that compares the favorable outcomes of the existing work with a single teacher using a teacher-centred and/or tactical games approach would be valuable. Moreover, the tactical games approach employed by the T-PT, as opposed to the teacher-centered pedagogy adopted by the U-PT, potentially impacted on the student recipient behaviors. However, the explicit impact of the individual components of the intervention strategy, such as the training, the resource-support, and the tactical games approach is unresolved.

Complementing greater ALT-PE, the trained peer-teachers provided more general feedback, positive feedback, and negative feedback during their periods of instruction. The appeal of being well placed to support others to learn was highlighted: “I thought it was good to give them feedback about the stuff … to tell them that they actually did better at something than they actually thought” (T-PT); and “I wrote down some notes and I told them, and they seem to have taken them in and actually improved from then on” (T-PT); and “It gave me a chance to go to them individually and talk to them on how they can improve…. after that, they have more confidence in themselves” (T-PT). While the provision of a teaching instructional manual was identified more frequently than the training as having a positive impact (see **Table 1**), it is not known if the T-PT were better placed to deliver feedback in response to an increased pedagogical content knowledge and/or as a result of enhanced confidence in their ability. Evidence to support the latter interpretation was apparent in some of the trained peer-teacher post-program comments: “…the training and booklet helped me to have more confidence and I knew what to talk about” (T-PT); and “…if there was something that I didn’t get, there was always a demonstration that I could look at so it [the instructional manual] was helpful” (T-PT); and

The training… made me ready for it, what to expect, what I needed to teach them and what I needed to do and encourage them and stuff. It [the program] has given me more confidence so I could actually talk to them and know what I had to do (T-PT).

The untrained peer-teachers reinforced the importance of the formalized teaching support: “It was a bit hard because I didn’t know what to teach exactly” (U-PT); and “Kind of figuring out what to do and we had to make it up” (U-PT), resulting in “... sometimes I asked them [the students] what they wanted to do” (U-PT).

Consistent with the higher levels of ALT-PE and feedback, the T-PT provided a significantly greater amount of structured learning time than those who did not receive training. Front-loading activities with goal-focused instructions and demonstrations, and/or the provision of within-task instructions, demonstrations, or augmented feedback that related to the defined learning outcomes was perceived to enhance students’ learning opportunities, as opposed to activities that were simply fun focused (i.e., unstructured lesson activities). Moreover, it is important to note that these additional learning opportunities and structure did not come at the expense of lower levels of student recipient physical activity. The instructional manual was highlighted by the T-PT as a significant facilitator of lesson structure: “It had all the information that we need, it didn’t just have a couple of points, it had every drill in it” (T-PT); and “The [instructional] book had diagrams which were useful, showed where the people were supposed to stand” (T-PT); and “… because, without them [the instructional manual] I wouldn’t really know where I am going and how I structure each lesson so that I don’t repeat the same thing” (T-PT). An untrained peer-teacher summed the challenges of creating learning-focused activities without the instructional manual: “I tried to start teaching them again and they just wanted to play games and stuff… they didn’t want to train anymore, just wanted to play.”

With the T-PT using a tactical games approach providing greater ALT-PE, feedback and structured learning time than their untrained counterparts, it was congruent that their peer-recipients displayed a significant improvement in their efficient/appropriate in-game performance actions over the course of the intervention (which was not apparent for those under U-PT). While these learning outcomes challenge recent concerns for the effectiveness of some peer teaching interventions (Jenkinson et al., 2013), they are consistent with previous work (Ernst and Byra, 1998; d’Arripe-Longueville et al., 2002; Ensergueix and Lafont, 2010; Iserbyt et al., 2011). It is also important to note, however, that student recipients in both groups displayed a significant increase in the number of inefficient/inappropriate actions observed between time 1 and time 2. Although the reason for this finding requires further investigation, students in both groups may have felt more confident and/or felt that they had a greater repertoire of skills following their soccer classes; thus encouraging them to attempt more demanding skills at time 2, or enabling them to implement stronger defensive and strategic plays against their opponents.

It is noteworthy that this was the first time a T-PT program has been implemented and evaluated, a point not lost on some of the trained peer-teachers: “it’s the first time for me so it was a good thing to experience, teaching things to people who are equal with me” (T-PT); and “… it gave me a chance to show and improve and get out there instead of not try anything. It makes me feel great” (T-PT); and “It’s been fun, never really done anything like it before” (T-PT). When interviewed, some of the T-PT also identified interpersonal outcomes in response to undertaking this innovative teaching approach:

I find that in sports now, even though I am not a PE teacher, I do tend to sometimes help other students when they are struggling. Even in class, I got a little bit more patient if they asked for help. I really enjoyed that and I would do it again (T-PT).

Moreover, some trained peer-teachers appeared enlightened: “I was thinking like now, of becoming a sports teacher because I enjoyed it quite a lot, … at the end, my teaching got a lot better” (T-PT); and “Well, it’s been very good, like the learning and wanting to be a teacher when I grow up” (T-PT).

In response to the findings the first hypothesis was accepted, confirming that T-PT using a tactical games approach displayed higher-quality teaching practices than U-PT who chose a teacher-centered pedagogy. The data confirmed a rejection of the second and third hypotheses whereby T-PT and U-PT reported similar PE motivation and enjoyment outcomes and elicited similar PA levels, PE enjoyment, and perception of the learning climate from their peer PE recipients. Students working under T-PT (as opposed to U-PT) did display greater motor skill (i.e., soccer performance) improvement from pre- to post-intervention, confirming acceptance of the fourth hypothesis.

The findings of this study should be considered within the context of potential limitations. Non-significant results for student recipient perceptions of PE enjoyment and the learning climate may suggest that the T-PT training sessions, supporting resource and the tactical games approach might not have been sufficient to significantly differentiate, in the eyes of some, the teaching and learning opportunities provided by the T-PT as opposed to the U-PT. To promote positive student outcomes, teachers need to have both an understanding of pedagogical and content knowledge (Dill, 1990). Whist it was verified through observation that the prescribed game-questions-practice-game sequence was used by the T-PT, the quality of the tactical games approach implementation was not evaluated. In addition, although this study engaged year 7 and 8 students (i.e., 11- to 13-years olds) the application to younger or older students may result in different outcomes. Future work that differentiates the impact of a T-PT program across gender, pre-unit motor ability levels, different sporting contexts, and in comparison to teacher-centered outcomes appears warranted. Indeed, the impact of grouping peer-teachers and students by gender or motor ability may be worthy of evaluation. Moreover, the timing, frequency, duration and focus of the training program are all elements that, with variation, may elicit different program outcomes. Students’ interview responses in this study indicate that future research into this pedagogy should also consider evaluating program outcomes related to enhancement of students’ satisfaction for relatedness support.

Although peer teaching has been lauded for its potential to enhance student learning (National Association for Sports and Physical Education [NASPE], 1995; Siedentop and Tannehill, 2000), this investigation offers an important qualification to that notion; specifically that peer teaching using student volunteers who (a) possess self-perceived context specific skills and motivation to teach their peers (non-reciprocal), and (b) receive formalized support through training and teaching resources underpinned by the tactical games approach, may engender important behavioral, pedagogical, and motor performance outcomes in PE.

## Conflict of Interest Statement

The authors declare that the research was conducted in the absence of any commercial or financial relationships that could be construed as a potential conflict of interest.
